# Identification of new genetic variants of HLA-DQB1 associated with human longevity and lipid homeostasis—a cross-sectional study in a Chinese population

**DOI:** 10.18632/aging.101323

**Published:** 2017-11-10

**Authors:** Fan Yang, Liang Sun, Xiaoquan Zhu, Jing Han, Yi Zeng, Chao Nie, Huiping Yuan, Xiaoling Li, Xiaohong Shi, Yige Yang, Caiyou Hu, Zeping Lv, Zezhi Huang, Chenguang Zheng, Siying Liang, Jin Huang, Gang Wan, Keyan Qi, Bin Qin, Suyan Cao, Xin Zhao, Yongqiang Zhang, Ze Yang

**Affiliations:** ^1^ The MOH Key Laboratory of Geriatrics, Beijing Hospital, National Center of Gerontology, Beijing, China; ^2^ Graduate School of Peking Union Medical College, Chinese Academy of Medical Sciences, Beijing, China; ^3^ Center for the Study of Aging and Human Development and Geriatrics Division, Medical School of Duke University, Durham, NC 27708, USA; ^4^ Center for Healthy Aging and Development Studies, National School of Development, Peking University, Beijing, China; ^5^ BGI-Shenzhen, Shenzhen, Guangdong, China; ^6^ Jiangbing Hospital, Guangxi Zhuang Autonomous Region, Nanning, Guangxi, China; ^7^ Office of Longevity Cultural, People's Government of Yongfu County, Yongfu, Guangxi, China; ^8^ Birth Defects Prevention and Control Research Institute, Guangxi Zhuang Autonomous Region Women and Children Health Care Hospital, Nanning, Guangxi, China; ^9^ Genetic Testing Center Qingdao Women and Children's Hospital, Qingdao University, Qingdao, China; ^10^ Department of Obstetrics and Gynecology, Aviation General Hospital of China Medical University, Beijing,China; ^11^ Capital Medical University Affiliated Beijing Ditan Hospital, Beijing, China

**Keywords:** HLA-DQB1, human longevity, lipid phenotypes, Chinese population

## Abstract

Healthy longevity has been an unremitting pursuit of human, but its genetic and the environment causes are still unclear. As longevity population is a good healthy aging model for understanding how the body begin aging and the process of aging, and plasma lipids metabolism and balance is a very important to life maintain and physiologic functional turnover. It is important to explore how the effect of genetic variants associated long-life individuals on lipids metabolism and balance. Therefore, we developed a comparative study based population which contains 2816 longevity and 2819 control. Through whole-exome sequencing and sanger sequencing genotypes, we identified four new single nucleotide polymorphisms of HLA-DQB1(major histocompatibility complex, class II, DQ beta 1), rs41542812 rs1049107 rs1049100 rs3891176(*P_range_*=0.048-2.811×10^−8^ for allele frequencies), associated with longevity in Chinese Longevity Cohort. Further, by analysis of the longevity-variants linked to blood lipids, we identified HLA-DQB1 rs1049107, T-carriers (*P_HDL_*=0.006, OR: 11.277; *P_TG_*=9.095×10^−7^, OR: 0.025; *P_LDL/HDL_*=0.047, OR: 1.901) and HLA-DQB1 rs1049100, T-carriers (*P_TG_*=1.799×10-6, OR: 0.028) associated with lipid homeostasis in long lived individuals. Our finding showed that longevity and lipid homeostasis were associated with HLA-DQB1 and suggested that immune gene variants could act on both new function of maintaining the homeostasis and anti-aging in longevity.

## INTRODUCTION

For thousands of years, humans have pursued increased longevity. However, increasing the longevity of the human population has been unlikely. In recent years, with the increase in social economic levels and an ageing population, the likelihood of an increase in human longevity is gradually increasing and is becoming the focus of people's attention. As we know, human longevity is determined by both genetic and environmental factors, but its genetics and the environ-ment causes, particularly their interaction mechanism, are still not clear. Therefore, it is necessary to develop the study and obtain supportive data for us to be able to provide inspiration and direction for future research in the field of longevity and anti-aging.

A long-lived population provides a good healthy ageing model for understanding how the body begins to age and the process of ageing. Several previous studies, both cross-sectional and follow-up, investigated long-lived subjects who usually manage to delay major age-related diseases, such as metabolic-related diseases (cardiovascular disease, diabetes, and neurodegenera-tion disorders); in general, these age-related diseases had high morbidity and mortality, but most centenarians escape these diseases [1-3] and successfully exhibit a healthy ageing state.

The common view is that human longevity is determined by genetic factors. Several Genome-wide association studies (GWAS) in European, North American and Chinese individuals identified some loci associated with longevity, i.e., TOMM40/APOE/APOC1 loci, 5q33.3, IL6 and ANKRD20A9P in recent years [4-6]. With next-generation sequencing (NGS), we have an efficient way to discover the potential causal loci in the search for sequence variants. NGS technology has facilitated the identification of variants and shown that HLA-DQB1*05 and HLA-DQB1*03 were associated with longevity in Japanese individuals [7, 8]. Previous studies of longevity genetics found that with increasing age, the contribution of heredity increased [9].

Along with ageing or senescence, the internal environment function of each hierarchy and each system is low and disordered, but there is still a lack of explicit evidence to define the genetically encoded program of ageing that functions to maintain the homeostasis of the human body, i.e., the plasma lipoproteins, in vivo.

The metabolism and balance of plasma lipids are very important to life and physiologic functional turnover. They are also the representative biomarker for cholesterol metabolism and lipid-related diseases such as cardiovascular and cerebrovascular diseases. A number of studies have shown that the plasma lipid levels were controlled by genetic factors and identified some associated variants of lipid levels, both common and rare [10-12]; for example, HLA-DQB1 variations were associated with plasma lipid balance in coronary heart disease risk [13], but there are no reports on genetic variants and plasma lipids associated with healthy longevity.

Therefore, we postulated that longer human survivors, who live to a median age of over 90 years, have a higher number of longevity genetic variants; briefly, ageing and longevity genetic variants serve as important biomarkers to represent the normal plasma lipid homeostasis in the internal environment of extremely elderly individuals. Therefore, based on our longevity cohort study in southern China, we developed a com-parative study using a case-control design to detect the relationship between longevity-associated variants and plasma lipid levels in our long-lived individuals in China. Our study aims to suggest that a healthy lipid balance linked to genetic variants in long-lived individuals could play an important part in human longevity, and our data will provide new knowledge to help us understand human longevity and the possible potential applications for anti-aging primary care in social communities of the elderly.

## RESULTS

### Baseline information in longevity and control subjects

There were significant differences in HDL-c (*P=*0.003), LDL-c (*P=*0.010), TG (*P=*3.052×10^−4^) and TC (*P=*0.012) between centenarians and controls. We detected that HDL-c (*P=*1.461×10^−7^), LDL-c (*P=*2.128×10^−4^) and TC (*P=*8.181×10^−6^) were sig-nificantly different between nonagenarians and controls. A comparison of longevity (centenarians and non-agenarians) in samples and controls, revealed that significant differences between HDL-c (*P=*3.438×10^−9^), LDL-c (*P=*1.269×10^−5^), TG (*P=*0.035) and TC (*P=*6.522×10^−7^) had been found (Supplementary Table 1).

### Identifying the new longevity-associated genetic variations

#### Whole-exome sequence screening in 100 longevity subjects

By whole exome sequence in 100 longevity subjects involving 74 nonagenarians and 26 centenarians, we compared both genomic differences between long-lived individuals and our genotype database of general Chinese (age <60 years, n=1000), and we primarily identified 2171 possible longevity-associated variants.

#### Bioinformatic and arrangement analysis

**Figure 1:**
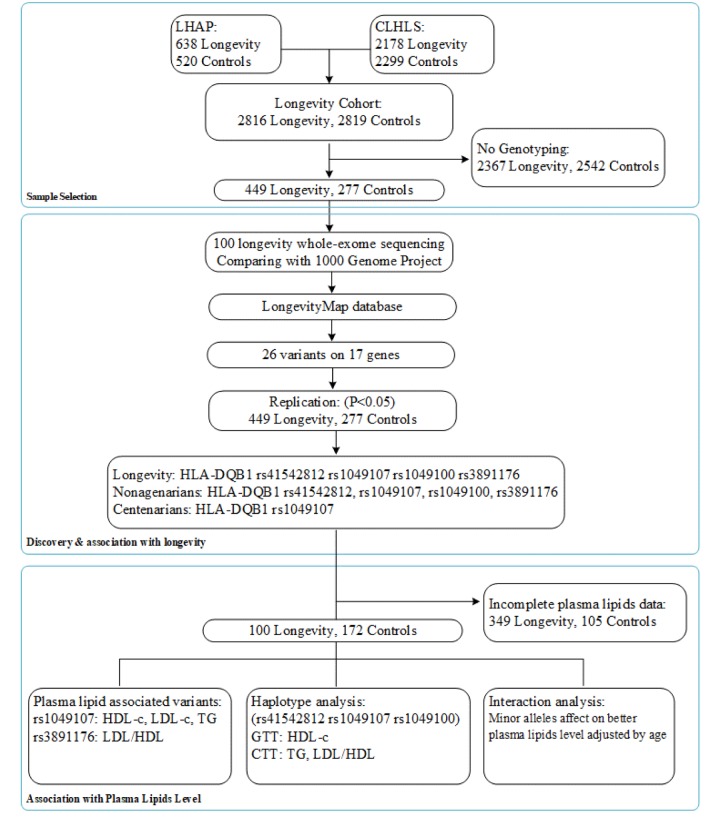
A flow chart of the consecutive analysis steps.

Aligning with GenBank, the analysis of the collection and arrangement of bioinformation based on the LongevityMap database [14] reported possible longevity variants, and we selected 17 genes with 26 variants as our longevity-associated candidate genes (Supplementary Table 2). A flow chart of the con-secutive analysis steps is depicted in Figure 1.

#### Identified new longevity-associated variants in HLA-DQB1

Through an association study based on genotyping longevity and a local younger population, we identified four genetic variants in HLA-DQB1 as longevity-associated gene variants. Four variants in HLA-DQB1 showed significant difference in allele and genotype frequencies between longevity and controls. These variants include rs41542812, rs1049107, rs1049100 and rs3891176, and this result was detected in a sample set what contained 518 longevities and 277 controls, rs41542812 (*P_allele_*=0.048, *P_genotype_*=0.003); rs1049107 (*P_allele_*=0.002, *P_genotype_*=0.007); rs1049100 (*P_allele_*=4.731×10^−4^, *P_genotype_*=0.002); and rs3891176 (*P_allele_*=2.811×10^−8^, *P_genotype_*=4.033×10^−8^ (Table 1).

**Table 1:** Association of variants and longevity

		Major allele	Minor allele				Major homo	Hetro	Minor home	
		Case/Control	Case/Control	P	OR	95%CI	Case/Control	Case/Control	Case/Control	P
Longevity vs. Controls	rs41542812	114/51	786/499	0.048	1.419	(1.001-2.011)	364/229	56/51	29/5	0.003
	rs1049107	199/86	653/440	0.002	1.559	1.178-2.064	278/190	97/60	51/13	0.007
	rs1049100	205/85	647/441	4.731×10^−4^	1.644	1.242-2.176	275/190	97/61	54/12	0.002
	rs3891176	248/84	600/440	2.811×10^−8^	2.165	1.642-2.854	253/186	94/68	77/8	4.033×10^−8^
Nonagenarians vs. Controls	rs41542812	48/51	262/499	0.006	1.793	1.176-2.732	123/229	16/51	16/5	6.652×10^−5^
	rs1049107	85/86	203/440	1.041×10^−5^	2.142	1.521-3.018	90/190	60/60	31/13	5.249×10^−7^
	rs1049100	89/85	199/441	9.297×10^−7^	2.320	1.650-3.264	88/190	23/61	33/12	9.258×10^−8^
	rs3891176	151/84	141/440	3.832×10-^27^	5.610	4.044-7.782	63/186	25/68	58/8	6.193×10^−21^
Centenarians vs. Controls	rs41542812	66/51	524/499	0.287	1.232	838-1.812	242/229	40/51	13/5	0.079
	rs1049107	114/86	450/440	0.001	1.296	0.951-1.766	188/190	74/60	20/13	0.317
	rs1049100	116/85	448/441	0.061	1.343	0.986-1.830	187/190	74/61	21/12	0.215
	rs3891176	107/84	449/440	0.166	1.248	0.911-1.710	190/186	69/68	19/8	0.131

#### Identified longevity-associated variation with ageing

**Figure 2:**
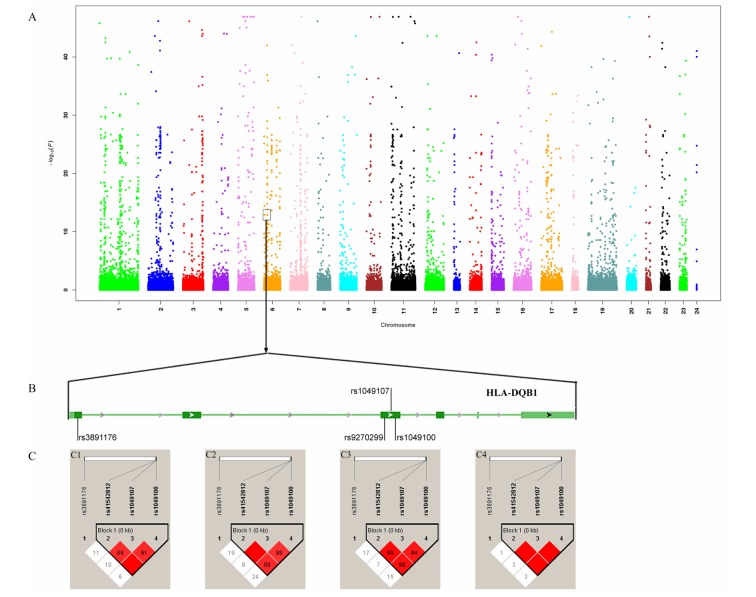
Association analysis identified HLA-DQB1 as longevity-associated gene (**A**) Manhattan map of whole-exome sequencing; (**B**) Four variants in HLA-DQB1; (**C**) Linkage disequilibrium analysis of four variants. C1 LD map of centenarians; C1 LD map of nonagenarians; C3: LD map of longevity; C4: LD map of controls.

We detected that rs41542812 (*P_allele_*=0.006, *P_genotype_*=6.652×10^−5^), rs1049107 (*P_allele_*=1.041×10^−5^, *P_genotype_*=5.249×10^−7^), rs1049100 (*P_allele_*=9.297×10^−7^, *P_genotype_*=9.258×10^−8^) and rs3891176 (*P_allele_*=3.832×10^−27^, *P_genotype_*=6.193×10^−21^) were associated with nonagenarians. Thus, rs1049107 (*P_allele_*=0.001, *P_genotype_*=0.317) was associated with centenarians (Table 1; Figure 2).

#### Identified longevity-associated haplotypes

The link disequilibrium analysis detected that there was a block, formed by rs41542812, rs1049107 and rs1049100, on HLA-DQB1 (Figure 2). There were three haplotypes with frequencies >0.03. Compared with the CCC haplotype, the GTT and CTT haplotypes could increase the chance of longevity (*P=*3.996×10^9^, OR: 4.367, 95%CI: 2.608-7.313; *P=*1.812×10^5^, OR: 3.677, 95%CI: 1.970-6.865). Comparing between centenarians and controls, the GTT and CTT haplotypes were associated with centenarians (*P=*3.327×10^8^, OR:6.484, 95%CI:3.128-13.440; and *P=*0.025, OR:3.373, 95%CI: 1.234-9.216, respectively). In nonagenarians, the GTT and CTT haplotypes would increase the chance to reach a nonagenarian age (*P=*1.921×10^6^, OR:3.721, 95%CI: 2.116-6.543; and *P=*3.604×10^5^, OR:3.770, 95%CI: 1.945-7.308, respectively; Supplementary Table 3).

### Relationship of longevity-associated variants and plasma lipid homeostasis

For rs1049107, Supplementary Table 4 shows the proportion of participants with different plasma lipid levels, normal or abnormal, and LDL-c/HDL-c ratios ≤2 and those >2, according to the polymorphism genotypes. The rs1049107 T allele was associated with a lower LDL-c/HDL-c ratio (*P=*0.047, OR: 1.901) in the longevity group.

In HDL-c subgroups of longevity samples, the rs1049107 TT genotype samples and T allele carriers showed a better HDL-c level than did the rs1049107 CC genotype samples (*P=*0.007, OR: 10.636; *P=*0.006, OR: 11.277). The normal HDL-c subgroup contained a higher rs1049107 T allele proportion (*P=*1.458×10^−4^, OR: 10.689), but this subgroup also had a lower rs3891176 C allele ratio than the major allele (*P=*0.013, OR: 0.360).

The combined rs1049107 genotype (TT+CT) in the control group, assuming a dominant effect of the T allele, was associated with a higher LDL-c level (*P=*1.243×10^−7^, OR: 0.098). In longevity group rs1049107 CT and rs1049100 CT, the genotype was associated with a higher TG level (*P=*9.095×10^−7^, OR: 0.025; *P=*1.799×10^−6^, 0.028), but it was opposite in the controls (*P=*0.004, OR: 11.667; *P=*0.004, OR: 11.667) compared with major homozygotes.

Overall, the dominant effect of the rs1049107 T allele was associated with a normal HDL-c level (*P=*0.001, OR: 72.757,95%CI: 4.378-984.360) and a higher TG level (*P=*0.035, OR: 0.312, 95%CI: 0.105-0.924) in the longevity group and with a higher LDL-c level in controls (*P=*0.050, OR: 0.383, 95%CI: 0.147-0.999) by a logistic regression analysis.

For the rs3891176 CA genotype, the dominant effect of the variant C allele and rs3891176 C allele were associated with a lower LDL-c/HDL-c ratio (Supplementary Table 3) by a logistic regression analysis, and the dominant effect of the rs3891176 C allele was associated with a lower LDL-c/HDL-c ratio (*P=*0.034, OR: 2.402, 95%CI: 1.068-5.402). Further-more, in the longevity group, we detected that major homozygotes of rs3891176 had significantly higher HDL-c levels compared to minor allele carriers (*P=*0.044) (Table 2).

**Table 2:** Allele and genotype distribution analysis in different plasma lipids level group

		Longevity						Control			
		HDL>1.04	HDL<=1.04	P	OR	95%CI	HDL>1.04	HDL<=1.04	P	OR	95%CI
rs41542812	CC	59	12	ref			98	29	ref		
	GC	3	1	0.541	0.610	0.058-6.377	14	8	0.175	0.518	0.198-1.356
	GG	20	1	0.285	4.068	0.497-33.288	3	0	1.000	1.212	0.13-11.262
	GG+GC	23	2	0.278	2.339	0.485-11.272	17	8	0.329	0.629	0.246-1.605
	CC+GC	62	13	0.291	0.238	0.029-1.939	112	37	1.000	0.743	0.081-6.858
	C	121	25	ref			210	66	ref		
	G	43	3	0.076	2.961	0.851-10.306	20	8	0.584	0.786	0.331-1.867
rs1049107	CC	44	13	ref			80	24	ref		
	CT	2	0	0.954	0.933	0.090-9.702	25	11	0.372	0.682	0.293-1.584
	TT	36	1	0.007	10.636	1.327-85.232	4	0	1.000	1.543	0.172-13.835
	TT+CT	38	1	0.006	11.227	1.403-89.846	29	11	0.579	0.791	0.345-1.815
	CC+CT	46	13	0.009	0.098	0.012-0.787	105	35	1.000	0.589	0.067-5.210
	C	90	26	ref			185	59	ref		
	T	74	2	1.458×10^−4^	10.689	2.456-46.523	33	11	0.907	0.957	0.455-2.011
rs1049100	CC	44	9	ref			80	24	ref		
	CT	1	1	0.333	0.205	0.012-3.583	25	11	0.372	0.682	0.293-1.584
	TT	37	4	0.314	1.892	0.539-6.646	4	0	1.000	1.543	0.172-13.835
	TT+CT	38	5	0.460	1.555	0.479-5.04	29	11	0.579	0.791	0.345-1.815
	CC+CT	45	10	0.247	0.486	0.141-1.678	105	35	1.000	0.589	0.067-5.210
	C	89	19	ref			185	59	ref		
	T	75	9	0.180	1.779	0.760-4.164	33	11	0.907	0.957	0.455-2.011
rs3891176	AA	57	7	ref			73	25	ref		
	CA	11	1	1.000	1.351	0.151-12.1	28	7	0.513	1.370	0.533-3.523
	CC	14	6	0.070	0.287	0.083-0.987	7	0	0.455	2.849	0.34-23.893
	CC+CA	25	7	0.219	0.439	0.139-1.383	35	7	0.253	1.712	0.676-4.34
	AA+CA	68	8	0.068	3.643	1.092-12.152	101	32	0.686	0.386	0.047-3.205
	A	125	15	ref			174	57	ref		
	C	39	13	0.013	0.360	0.158-0.822	42	7	0.116	1.966	0.837-4.618
		LDL<3.12	LDL>=3.12	P	OR	95%CI	LDL<3.12	LDL>=3.12	P	OR	95%CI
rs41542812	CC	44	27	ref			105	22	ref		
	GC	4	0	0.406	3.111	0.345-28.029	19	3	1.000	1.327	0.361-4.876
	GG	15	6	0.427	1.534	0.531-4.433	3	0	1.000	0.868	0.093-8.13
	GG+GC	19	6	0.204	1.943	0.690-5.472	22	3	0.512	1.537	0.423-5.587
	CC+GC	48	27	0.526	0.711	0.247-2.048	124	25			
	C	92	54	ref			229	47	ref		
	G	34	12	0.175	1.663	0.794-3.482	25	3	0.592	1.710	0.496-5.898
rs1049107	CC	34	23	ref			89	15	ref		
	CT	2	0	1.000	2.057	0.202-20.975	28	8	0.276	0.590	0.226-1.537
	TT	27	10	0.186	1.826	0.744-4.483	3	1	0.479	0.506	0.049-5.188
	TT+CT	29	10	0.136	1.962	0.804-4.789	31	9	0.244	0.581	0.231-1.46
	CC+CT	36	23	0.230	0.580	0.237-1.418	117	23			
	C	70	46	ref			206	38	ref		
	T	56	20	0.057	1.840	0.978-3.461	34	10	0.626	1.219	0.55-2.703
rs1049100	CC	32	21	ref			89	15	ref		
	CT	2	0	1.000	2.000	0.195-20.486	28	8	0.276	0.590	0.226-1.537
	TT	29	12	0.297	1.586	0.665-3.783	3	1	0.479	0.506	0.049-5.188
	TT+CT	31	12	0.229	1.695	0.714-4.024	31	9	0.244	0.581	0.231-1.46
	CC+CT	34	21	0.363	0.670	0.282-1.591	117	23			
	C	66	42	ref			206	38	ref		
	T	60	24	0.135	1.591	0.863-2.932	34	10	0.626	1.219	0.55-2.703
rs3891176	AA	43	21	ref			79	19	ref		
	CA	7	5	0.741	0.684	0.194-2.412	32	3	0.139	2.565	0.71-9.273
	CC	13	7	0.856	0.907	0.315-2.609	4	3	0.158	0.321	0.066-1.555
	CC+CA	20	12	0.649	0.814	0.336-1.974	36	6	0.470	1.443	0.531-3.918
	AA+CA	50	26	1.000	1.036	0.368-2.912	111	22			
	A	93	47	ref			190	41	ref		
	C	33	19	0.700	0.878	0.452-1.706	40	9	0.918	0.959	0.432-2.13
		TG<1.7	TG>=1.7	P	OR	95%CI	TG<1.7	TG>=1.7	P	OR	95%CI
rs41542812	CC	58	13	ref			89	38	ref		
	GC	3	1	0.738	0.672	0.065-6.993	13	9	0.306	0.617	0.243-1.565
	GG	17	4	0.939	0.953	0.275-3.306	3	0	1.000	1.733	0.188-16.012
	GG+GC	20	5	1.000	0.897	0.284-2.831	16	9	0.531	0.751	0.305-1.847
	CC+GC	61	14	0.287	0.256	0.031-2.090	102	47			
	C	119	27	ref			191	85	ref		
	G	37	9	0.871	0.933	0.403-2.16	19	9	0.883	0.939	0.408-2.162
rs1049107	CC	50	7	ref			70	34	ref		
	CT	2	11	9.095×10^−7^	0.025	0.005-0.140	23	0	0.004	11.667	1.516-89.79
	TT	26	0	0.153	4.235	0.503-35.659	4	13	0.001	0.149	0.045-0.493
	TT+CT	28	11	0.050	0.356	0.124-1.023	27	13	0.982	1.009	0.463-2.197
	CC+CT	52	18	0.010	0.103	0.013-0.814	93	34			
	C	102	25	ref			163	68	ref		
	T	54	11	0.643	1.203	0.550-2.63	31	26	0.020	0.497	0.275-0.9
rs1049100	CC	46	7	ref			70	34	ref		
	CT	2	11	1.799×10^−6^	0.028	0.005-0.152	23	0	0.004	11.667	1.516-89.79
	TT	30	0	0.146	5.277	0.628-44.302	4	13	0.001	0.149	0.045-0.493
	TT+CT	32	11	0.122	0.443	0.155-1.264	27	13	0.982	1.009	0.463-2.197
	CC+CT	48	18	0.003	0.083	0.011-0.653	93	34			
	C	94	25	ref			163	68	ref		
	T	62	11	0.306	1.499	0.688-3.264	31	26	0.020	0.497	0.275-0.9
rs3891176	AA	49	15	ref			65	33	ref		
	CA	11	1	0.442	3.367	0.401-28.258	26	9	0.385	1.467	0.617-3.487
	CC	18	2	0.338	2.755	0.573-13.259	5	2	1.000	1.269	0.234-6.896
	CC+CA	29	3	0.096	2.959	0.789-11.099	31	11	0.382	1.431	0.639-3.201
	AA+CA	60	16	0.347	0.417	0.087-1.986	91	42			
	A	109	31	ref			156	75	ref		
	C	47	5	0.048	2.673	0.979-7.3	36	13	0.416	1.331	0.667-2.658
		TC<5.2	TC>=5.2				TC<5.2	TC>=5.2			
rs41542812	CC	45	26	ref			113	14	ref		
	GC	4	0	0.661	2.411	0.268-21.661	19	3	0.719	0.785	0.206-2.992
	GG	15	6	0.496	1.444	0.499-4.181	3	0	0.476	0.526	0.055-5.026
	GG+GC	19	6	0.250	1.830	0.649-5.161	22	3	1.000	0.909	0.241-3.428
	CC+GC	49	26	0.600	0.754	0.261-2.175	132	17			
	C	94	52	ref			245	31	ref		
	G	34	12	0.232	1.567	0.748-3.286	25	3	1.000	1.054	0.301-3.697
rs1049107	CC	36	21	ref			94	10	ref		
	CT	2	0	1.000	1.784	0.175-18.222	30	6	0.360	0.532	0.178-1.586
	TT	26	11	0.477	1.379	0.568-3.347	4	0	0.502	0.579	0.062-5.416
	TT+CT	28	11	0.378	1.485	0.615-3.583	34	6	0.381	0.603	0.204-1.785
	CC+CT	38	21	0.553	0.766	0.316-1.853	124	16	0.547	1.471	0.162-13.352
	C	74	42	ref			218	26	ref		
	T	54	22	0.297	35.000	0.747-2.6	38	6	0.602	0.755	0.291-1.957
rs1049100	CC	34	19	ref			94	10	ref		
	CT	2	0	1.000	1.714	0.167-17.6	30	6	0.360	0.532	0.178-1.586
	TT	28	13	0.674	1.204	0.507-2.858	4	0	0.502	0.579	0.062-5.416
	CC+CT	36	19	0.770	0.880	0.372-2.081	124	16	0.547	1.471	0.162-13.352
	TT+CT	30	13	0.562	1.290	0.546-3.046	34	6	0.381	0.603	0.204-1.785
	C	70	38	ref			218	26	ref		
	T	58	26	0.537	1.211	0.659-2.225	38	6	0.602	0.755	0.291-1.957
rs3891176	AA	41	23	ref			86	12	ref		
	CA	8	4	1.000	1.122	0.304-4.135	33	2	0.354	2.302	0.489-10.845
	CC	15	5	0.365	1.683	0.542-5.229	6	1	1.000	0.837	0.093-7.567
	CC+CA	23	9	0.444	1.434	0.569-3.613	39	3	0.553	1.814	0.484-6.794
	AA+CA	49	27	0.552	0.726	0.252-2.089	119	14	0.556	1.417	0.159-12.636
	A	90	50	ref			205	26	ref		
	C	38	14	0.251	1.508	0.746-3.047	45	4	0.525	1.427	0.474-4.291
		LDL/HDL≤2	LDL/HDL>2	P	OR	95%CI	LDL/HDL		P	OR	95%CI
rs41542812	CC	47	24	ref			71	56	ref		
	GC	3	1	1.000	1.532	0.151-15.526	12	10	0.906	0.946	0.381-2.35
	GG	14	7	0.968	1.021	0.364-2.866	2	1	0.710	1.577	0.139-17.845
	GG+CC	17	8	0.869	1.085	0.410-2.873	14	11	0.993	1.004	0.423-2.381
	CC+GC	50	25	1.000	1.000	0.358-2.791	83	66	0.705	0.629	0.056-7.086
	C	97	49	ref			154	122	ref		
	G	31	15	0.905	1.044	0.515-2.114	16	12	0.891	1.056	0.482-2.316
rs1049107	CC	35	22	ref			61	43	ref		
	CT	1	1	1.000	0.629	0.037-10.573	16	20	0.140	0.564	0.263-1.211
	TT	28	9	0.150	1.956	0.778-4.912	2	2	1.000	0.705	0.096-5.201
	TT+CT	29	10	0.186	1.823	0.745-4.461	18	22	0.140	0.577	0.277-1.203
	CC+CT	36	23	0.208	0.503	0.201-1.257	77	53	1.000	1.453	0.198-10.638
	C	71	45	ref			138	106	ref		
	T	57	19	0.047	1.901	1.003-3.604	20	24	0.173	0.640	0.336-1.22
rs1049100	CC	34	19	ref			61	43	ref		
	CT	1	1	1.000	0.559	0.033-9.451	16	20	0.140	0.564	0.263-1.211
	TT	29	12	0.501	1.350	0.562-3.244	2	2	1.000	0.705	0.096-5.201
	TT+CT	30	13	0.562	1.290	0.546-3.046	18	22	0.140	0.577	0.277-1.203
	CC+CT	35	20	0.466	0.724	0.304-1.726	77	53	1.000	1.453	0.198-10.638
	C	69	39	ref			138	106	ref		
	T	59	25	0.355	1.334	0.724-2.457	20	24	0.173	0.640	0.336-1.22
rs3891176	AA	44	20	ref			49	49	ref		
	CA	6	6	0.209	0.455	0.130-1.585	26	9	0.013	2.889	1.228-6.794
	CC	14	6	0.916	1.061	0.356-3.163	4	3	0.715	1.333	0.283-6.272
	CC+CA	20	12	0.540	0.758	0.311-1.844	30	12	0.019	2.500	1.149-5.442
	AA+CA	50	26	0.795	0.824	0.283-2.396	75	68	1	0.827	0.179-3.830
	A	94	46	ref			124	107	ref		
	C	34	18	0.818	0.924	0.472-1.809	34	15	0.044	1.956	1.011-3.785

### Analysis of plasma-lipid homeostasis and longevity-associated haplotypes

To analyse the effect of haplotypes on plasma lipids, we divided samples between plasma lipid levels and age. We found that rs41542812, rs1049107 and rs1049100 would form a block in HLA-DQB1 and that the CCC haplotype, which was formed by major alleles, seemed to be a reference. In the centenarian group, we obtained none of the significant haplotypes for four plasma lipid indices. In nonagenarians, the block contained three haplotypes, and the CTT haplotype was associated with a higher LDL-c/HDL-c ratio (*P=*0.037, OR: 0.301, 95%CI: 0.093-0.969). In the longevity group, the GTT and CTT haplotypes were associated with higher HDL-c levels (*P=*0.027, OR:4.750, 95%CI: 1.057-21.337; *P=*0.045, OR:6.667, 95%CI: 0.857-51.883). The CTT haplotype was associated with a higher TG level (*P=* 1.096×10^−4^), OR: 0.178, 95%CI: 0.070-0.451), and the CTT haplotype was associated with LDL-c/HDL-c <2 (*P=*0.027, OR: 3.403, 95%CI: 1.098-10.544; Supplementary Table 5).

### Interaction between longevity-associated variants and internal milieu lipid levels

An interaction analysis between longevity associated variants and internal milieu lipid levels on age detected significant results in a corrected model (R^2^=0.375, R^2^_Adjusted_=0.332). Significant values of rs14917 & rs1491399 (*P=*0.001), rs1491 & LDL-c/HDL-c (*P=*0.016), rs927299 & LDL-c/HDL-c (*P=*0.028), and rs1491 & rs927299 & LDL-c/HDL-c (*P=*0.031) were detected in this model.

## DISCUSSION

### Longevity-associated variants and haplotypes identified

Because of the low prevalence of delayed age-related disease, the longevity population was an ideal population to be considered a “real negative control” in human successful healthy ageing and age-related disease. Genetic research about longevity had identify some longevity-associated factors, such as FOXO3A [15, 16], SIRT1 [17], APOE [18, 19], IL6 and ANKRD20A9P [20]. There had been multiple genetic studies of Chinese longevity, and FOXO3 [21], IGFBP-3 [22], CETP [23], and SIRT1 [24]. Studies in Sardinian centenarians showed the association between HLA-DQB1 and longevity [25, 26]. The association between longevity and HLA-DQB1*05 or HLA-DQB1*03 was identified in a Japanese population [7, 8]. However, there was no association study about SNPs in HLA-DQB1 and longevity. In this study, we identified four new SNPs in HLA-DQB1, rs41542812 (DQB1*05), rs1049107 (DQB1*03), rs1049100 (DQB1*03) and rs3891176 (DQB1*02), associated with longevity in our cohort (Table 1). Further, we identified that rs41542812, rs1049107, and rs1049100 were closely linked to disequilibrium in one block; that is, the haplotypes GTT and CTT, which significantly increased the chance of longevity (*P=*3.996×10^9^, OR: 4.367, 95%CI: 2.608-7.313; *P=*1.812×10^5^, OR: 3.677, 95%CI: 1.970-6.865) to our knowledge have also never been reported before.

In these new longevity-associated variants, we noted through the use of stratified analysis that only rs1049107 was a centenarian-associated variant in long-lived individuals, but the other three variants were associated with both centenarians and nonagenarians (Table 1). This result suggested that the longevity-associated genetic variance seems to increase with ageing, so we observed the variance only in centena-rians but not nonagenarians.

### Longevity variants associated with lipid homeostasis in LLIs

The main cholesterol-carrying lipoproteins are LDL-c and HDL-c. LDL-c is always considered the bad lipo-protein and can increase CVD and metabolic syndrome risk. In contrast, HDL-c is considered the good lipoprotein and can decrease metabolic-related disease and CVD risk. The importance of the balance of plasma lipids for maintaining health is obvious. The LDL-c/HDL-c ratio is increasingly being considered a valuable tool in CVD-related clinical research. Many variants have been identified as potentially affecting human lipids, as have some longevity-associated variants. The changes in lipid levels in our cohort were consistent with those in previous studies [27-29]. However, evidence for the balance of plasma lipids being controlled by longevity-associated loci is still lacking.

Furthermore, we identified HLA-DQB1 rs1049107, T-carriers (=0.006, OR: 11.277; P_TG_=9.095×10^−7^, OR: 0.025; P_LDL/HDL_=0.047, OR: 1.901) and HLA-DQB1 rs1049100, T-carriers (P_TG_=1.799×10^−6^, OR: 0.028) associated with lipid homeostasis in LLIs.

### The interaction analysis of longevity variants and lipid levels

The interaction analysis between lipids and genotype results show that HLA-DQB1 longevity variant alleles or haplotype carriers have a trend of better blood lipid levels, thus benefiting healthy longevity in LLIs (Table 3; Supplementary Figure 1). The interaction analysis results suggest that with longevity-associated variants enriched in the longevity population, the plasma lipid levels were better controlled than by adjusting age. They also suggested that it is possible for longevity variants to possess functions to regulate the homeostasis of plasma lipids in the internal environment of LLIs.

**Table 3:** The effect of variants and lipid levels interaction on long-life (years) in all subjects

Source	Type III Sum of Squares	df	Mean Square	F	P
Corrected Model	104143.199	32	3254.475	8.738	<0.001
	8045.243	1	8045.243	21.602	<0.001
rs14917*rs1491399	3.124	1	3993.124	10.547	0.001
rs1491*LD/LHDL	2164.922	1	2164.922	5.813	0.016
rs927299*LDL/HDL	2692.908	2	1346.454	3.615	0.028
rs1491*rs927299*LDLHDL	6703.210	8	837.901	2.136	0.031
Error	173924.649	467	372.430		
Total	2559434.000	500			
Corrected Total	278067.848	499			

### Functional analysis of the new longevity-associated variants in HLA-DQB1

**Figure 3:**
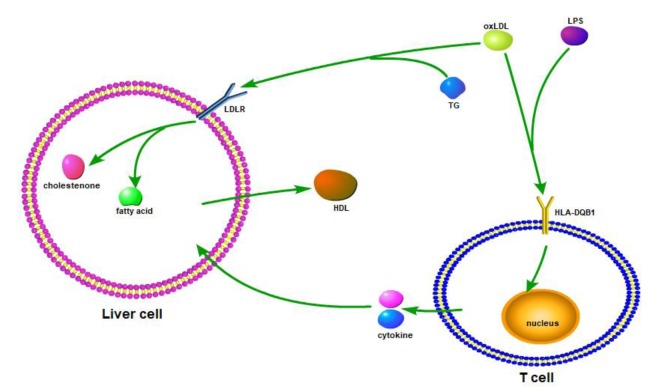
Based on the resource from relevant information on HLA-DQB1 and homeostasis/metabolism phenotype, we draw the possible mechanic or interactive path way.

HLA-DQB1 contained 5 exons. Exon 1 encodes the leader peptide, exons 2 and 3 encode the two extracellular protein domains, exon 4 encodes the transmembrane domain, and exon 5 encodes the cyto-plasmic tail. All four novel variants in HLA-DQB1 are functional missense mutations. I These four variants, rs41542812 (Gln158His), rs1049107 (Gly157Ser) and rs1049100 (Val148Ile), lays in β chain of HLA-DQB1. Variety in β chain may change the binding site for special antigen, which plays a key role in maintaining internal homeostasis in vivo, such as lipid homeostasis. In addition, rs3891176 (Ala29Ser), located in exon 1, may change the function of the leader peptide and transform the signal that HLA-DQB1 received (Figure 3). Therefore, we first identified the association between longevity and these four variants. oxLDL (oxidized low-density lipoprotein) or LPS bands on HLA-DQB1 of T cell, and stimulate T cell producing cytokines. Cytokines effect on liver cell, and increase or decrease fatty acid metabolism. OxLDL could also bands on LDLR (low-density lipoprotein receptor) or HLA-DQB1 of liver cell. The signal could also change fatty acid metabolism and HDL-c producing of liver cell. With this possible process, plasma lipid homeostasis could affect by HLA-DQB1 variants (Figure 3).

We speculated that the HLA-DQB1 variants decreased the expression and/or antigen binding function of the HLA Class II antigen DQ protein β chain on the Antigen Presenting Cell (i.e., the macrophage), reduces the cytokines released by T-lymphocytes, thus reducing liver cell synthesis and release of cholesterol, to maintain the balance of cholesterol metabolism in vivo. This theory remains to be studied more in depth to obtain the corresponding evidence.

Findings in this research suggest a possible gene effecting model. HLA-DQB1 affects the longevity through a certain biological pathway, and these pathways could influence the balance of plasma lipids through unknown biological process. Longevity and the balance of plasma lipids could affect each other. A longevity gene could help keep plasma lipids balanced, and a better balance of plasma lipids could promote homeostasis and healthy longevity. However, the mechanisms of longevity and plasma lipid levels are still not clear. Furthermore, a gene associated with longevity and plasma lipid levels could be identified in multiple large longevity populations, and rare or low-frequency variants must receive more attention. The mechanism could be studied in cell or animal models by using new technology, such as Crispr/Cas 9. It would be useful for us to understand the mechanism of longevity and help the wider community achieve healthy ageing.

## METHODS

### Subjects

The current study was based on the Longevity and Health of Aging Population (LHAP) study conducted in Bama County, Guangxi, China, in 2008 and the Chinese Longitudinal Healthy Longevity Survey (CLHLS) during 1997-2015 (Supplementary Table 6).

To investigate the association between genotype and plasma lipid levels, there were 100 long-lived individuals (aged 90-107 years, 32 males, 68 females), and 172 controls (aged 40-65 years, 75 males, 97 females). The longevity group contained 26 centena-rians (5 males, 21 females) and 74 nonagenarians (27 males, 47 females). All control subjects lacked longevity history (no lineal family members aged above 85 for three generations). The Ethics Committee of Beijing Hospital, Ministry of Health, approved the study protocol. All participants were informed and provided informed consent in writing. All clinical investigations were conducted according to the principles of the Declarations of Helsinki. Laboratory parameters were recorded, including total cholesterol (TC), triglyceride (TG), high density lipoprotein-cholesterol (HDL-c) and low-density lipoprotein-cholesterol (LDL-c).

### Exome target sequencing

We sequenced the segment of exomes using a targeted NGS approach to analyse SNVs (single nucleotide variations) and small insertions and/or deletions (Indels). The design parameters were as follows: 1) the bait length was 120 bp; 2) the bait tiling frequency was 2×; and 3) the human reference genome (GRch37) was used to search the baits. Genomic DNA (3 μg) was used to construct DNA libraries that contained index sequences for identifying DNA samples. The targeted genomic segment was captured with the Agilent Capture kit 39M (Agilent Technologies, Santa Clara, CA, USA) by NGS on the Illumina HiSeq 2500 platform to identify mutations in 100 Bama longevity subjects using 100-bp paired-end reads. Base sequences were aligned to the reference genome (GRch37), and variations SNVs and Indels) were called using the Samtools software and annotated by comparing them with the 1000 Genomes database. The average coverage of each base in the targeted region was 50×, which resulted in ≥90% of the targeted bases being sufficiently covered for variant calling (≥10×). The average coverage was ≥95% at the NGS stage. We compared the data from our 100 LLIs to publicly available genotype data from a CHS (Chinese Han South) population (n = 97) from the 1000 Genome project phase I. We found 115327 SNVs, in total. Of the 115327 SNVs, 84914 were consistent with those in the public SNP database, and 30413 were previously unknown.

### Selection of variations

We selected candidate variation from the target sequence dataset using four continuous steps.

1) To minimize error rates and the impact of random sampling variance, at least three reads had to be obtained from both the forward and reverse DNA strands (double-stranded coverage) for all variants that were kept for association tests.

2) Variants located at repeat sequences, including STR (short tandem repeat) or single nucleotide repeat expansions, may be falsely called; thus, we first excluded these possible artificial variants.

3) The Longevity Gene database was downloaded, and all significant genes were screened. All variants of these significant genes were called and compared with the CHS population in the 1000 Genome project to obtain candidate variants with MAF (minor allele frequency) >10% in the 100 Bama longevity subjects compared to those in one public database. Seventeen genes that includ-ed 26 variants were selected as candidate longevity-associated genes. Through genotyping based on longevity and local younger populations, four genetic variants in HLA-DQB1 were identified as longevity-associated gene variants (Supplementary Table 1).

### Genotyping and quality control for genotyping

The Sanger Sequencing method was used in the genotyping case-control study. To perform genotyping quality control, all associated allele carriers, and 10% of cases and 10% of controls who carried non-risk alleles, were re-genotyped by Sanger sequencing. Sequencing primers are shown in Supplementary Table 7.

### Statistical analysis

Genotypes were evaluated for departure from the Hardy–Weinberg equilibrium (HWE) in cases and controls using the chi-squared goodness test. Variants with P < 0.05 were considered to deviate from the HWE. The MAFs of the variants were used as high-risk allele frequencies, and the defined type I error rate was 0.05. The genotype frequencies of the CHS population from the 1000 Genome database were used as the references for selecting candidate SNVs. A t-test was used to analyse the mean difference between the groups. A chi-squared test was used to establish the differences in the distribution of genotype and allele frequencies between the cases and controls. The odds ratio (OR) was used to estimate the strength of association between variables, with the OR and 95% confidence intervals (95%CI). The ORs and 95% CIs were calculated using the SPSS V17.0 software (SPSS; SAS Institute, Cary, NC, USA). A two-sided P value < 0.05 was considered statistically significant.

## CONCLUSIONS

In this research, we identified four longevity-associated variants in HAL-DQB1, and these variants were associated with different kinds of plasma lipids. Our finding shows that longevity and plasma lipid levels were affected by multiple genes and that internal lipid homeostasis promoted healthy longevity. Further similar studies should be developed in human populations, especially investigating human lifespan and healthy longevity.

## SUPPLEMENTARY MATERIAL FIGURE AND TABLES


