# Correction: Identification of new genetic variants of HLA-DQB1 associated with human longevity and lipid homeostasis—a cross-sectional study in a Chinese population

**DOI:** 10.18632/aging.101400

**Published:** 2018-03-15

**Authors:** Fan Yang, Liang Sun, Xiaoquan Zhu, Jing Han, Yi Zeng, Chao Nie, Huiping Yuan, Xiaoling Li, Xiaohong Shi, Yige Yang, Caiyou Hu, Zeping Lv, Zezhi Huang, Chenguang Zheng, Siying Liang, Jin Huang, Gang Wan, Keyan Qi, Bin Qin, Suyan Cao, Xin Zhao, Yongqiang Zhang, Ze Yang

**Affiliations:** 1 The MOH Key Laboratory of Geriatrics, Beijing Hospital, National Center of Gerontology, Beijing, China; 2 Graduate School of Peking Union Medical College, Chinese Academy of Medical Sciences, Beijing, China; 3 Center for the Study of Aging and Human Development and Geriatrics Division, Medical School of Duke University, Durham, NC 27708, USA; 4 Center for Healthy Aging and Development Studies, National School of Development, Peking University, Beijing, China; 5 BGI-Shenzhen, Shenzhen, Guangdong, China; 6 Jiangbing Hospital, Guangxi Zhuang Autonomous Region, Nanning, Guangxi, China; 7 Office of Longevity Cultural, People's Government of Yongfu County, Yongfu, Guangxi, China; 8 Birth Defects Prevention and Control Research Institute, Guangxi Zhuang Autonomous Region Women and Children Health Care Hospital, Nanning, Guangxi, China; 9 Genetic Testing Center Qingdao Women and Children’s Hospital, Qingdao University, Qingdao, China; 10 Department of Obstetrics and Gynecology, Aviation General Hospital of China Medical University, Beijing,China; 11 Capital Medical University Affiliated Beijing Ditan Hospital, Beijing, China

Original article: Aging (Albany NY) 2017; 9(11): 2316-2333

PMCID: PMC PMC5723689 PMID: 29129831 doi: 10.18632/aging.101323

Please check the link to the original paper: http://www.aging-us.com/article/101323/text

**Present:** Due to the author’s mistake, the affiliation 2 is not correct

**Corrected:** The correct affiliation is: Graduate School of Peking Union Medical College, Chinese Academy of Medical Sciences, Beijing, China

The authors sincerely apologize for this error.

