# Supplementary material for: Identification of new genetic variants of HLA-DQB1 associated with human longevity and lipid homeostasis—a cross-sectional study in a Chinese population
Source: Aging (Albany NY). 2017 Nov 10;9(11):2316–33. doi: 10.18632/aging.101323 (PMC5723689; doi:10.18632/aging.101323)
Supplement: Supplementary file 1 [file aging-09-2316-s001.pdf]

SUPPLEMENTARY MATERIAL

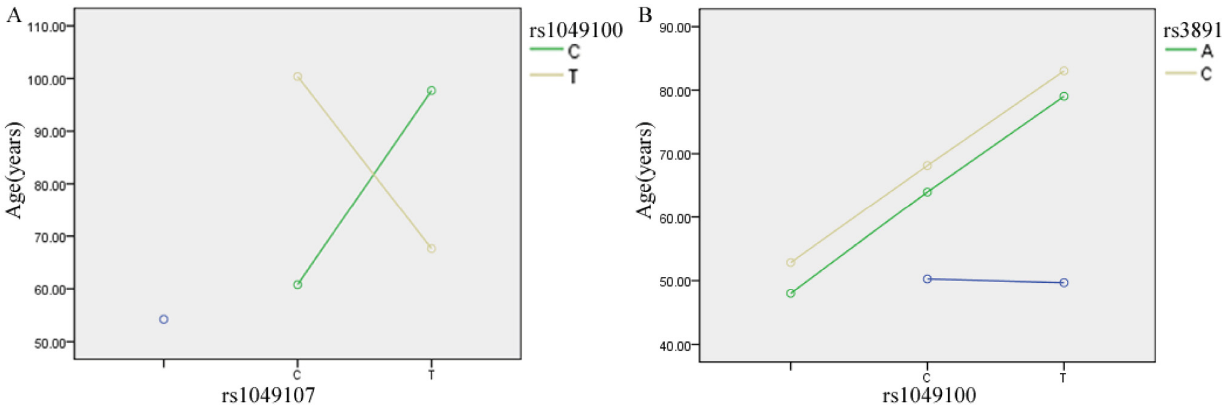

Supplementary Figure 1. Interaction analysis between plasma lipids level and variants on age. HLA-DQB1 longevity variant allele carriers take a trend of better blood lipid homeostasis A: interaction analysis between rs1049107 and rs1049100 B: interaction analysis between rs149107, rs3891176 and LDL/HDL.

Supplementary Table 1. Base line of plasma lipids in different age groups.

	N (Centenarians)	Mean±Std	N (Nonagenarians)	Mean±Std	N (Controls)	Mean±Std	N (Longevity)	Mean±Std	P(Longevity VS. Control)	P(LongevityVS Controls)	P(Centenarians VS Nonagenarians)
HDL	25	1.548±0.463	71	1.543±0.421	154	1.230±0.301	96	1.544±0.430	0.003	3.438×10^{-9}	0.963
LDL	25	2.992±1.088	71	2.931±1.125	154	2.370±0.733	96	2.947±1.110	0.010	1.269×10^{-5}	0.815
TG	25	0.957±0.383	71	1.409±1.042	154	1.645±1.462	96	1.291±0.936	3.052×10^{4}	0.035	0.003
TC	25	4.731±1.452	71	4.756±1.345	154	3.928±0.923	96	4.749±1.366	0.012	6.522×10^{-7}	0.939
LDL/HDL	25	1.975±0.625	71	1.977±0.726	154	2.060±1.098	96	1.977±0.698	0.707	0.508	0.988

Supplementary Table 2. Information of candidate longevity-associated variants.

Chrosome	Gene	Position	ID	Alleles	Variant_MAF	CHS-MAF	Function
chr2	XDH	31571786	rs1884725	A/G	0.05	0.12	synonymous
chr5	CSF1R	149457678	rs2228422	G/A	0.48	0.17	synonymous
chr6	HLA-DRB1	32557435	rs9270299	A/C	0.32	0.14	missense
chr6	HLA-DQA1	32605284	rs12722039	G/A	0.31	0.10	missense
chr6	HLA-DQA1	32605309	rs12722042	A/G	0.31	0.10	missense
chr6	HLA-DQA1	32610461	rs9260	A/G	0.46	0.19	missense
chr6	HLA-DQB1	32629931	rs41542812	C/G	0.42	0.10	missense
chr6	HLA-DQB1	32629936	rs1049107	C/T	0.42	0.18	missense
chr6	HLA-DQB1	32629963	rs1049100	C/T	0.45	0.18	missense
chr6	HLA-DQB1	32666541	rs3891176	A/C	0.19	0.05	missense
chr6	FOXO3	108977663	rs9400239	T/C	0.23	0.19	UTR-5
chr6	SKG1	134493397	rs1057293	G/A	0.43	0.19	synonymous
chr6	IGF2R	160453978	rs1570070	A/G	0.17	0.24	synonymous
chr10	ADARB2	1717343	rs12258319	G/T	0.17	0.08	intron
chr11	DRD4	640119	rs762502	C/T	0.49	0.23	synonymous
chr13	CPB2	46629944	rs1926447	A/G	0.14	0.20	missense
chr15	RYR3	34113536	rs2288614	A/G	0.09	0.20	synonymous
chr16	MEFV	3293888	rs1231122	C/T	0.30	0.31	missense
chr16	MEFV	3294246	rs77380520	G/A	0.34	0.08	intron
chr16	MEFV	3297100	rs76464258	G/A	0.34	0.08	synonymous
chr16	MEFV	3297181	rs224208	C/T	0.17	0.30	synonymous
chr19	MYO9B	17273893	rs7256689	G/T	0.10	0.20	synonymous
chr19	TOMM40	45404691	rs405697	A/G	0.31	0.32	synonymous
chr19	APOE	45409167	rs440446	C/G	0.30	0.33	intron
chr21	ADARB1	46644563	rs11701974	A/G	0.48	0.28	UTR-3
chr21	ADARB1	46644599	rs11701976	A/G	0.32	0.28	UTR-3

Supplementary Table 3. Haplotype analysis of rs41542812 rs1049107 and rs1049100.

Haplotype	Longevity	Control	P	OR	95%CI
CCC	107	267			
GTT	49	28	3.997×10^{-9}	4.367	2.608-7.313
CTT	28	19	1.812×10^{-5}	3.677	1.970-6.865
	Centenarians	Control			
CCC	25	267			
GTT	17	28	3.327×10^{-8}	6.484	3.128-13.440
CTT	6	19	0.025	3.373	1.234-9.216
	Nonagenarians	Control			
CCC	82	267			
GTT	32	28	1.921×10^{-6}	3.721	2.116-6.543
CTT	22	19	3.604×10^{-5}	3.770	1.945-7.308

Supplementary Table 4. Association between genotype and plasma lipids level in different group.

	Longevity								Control							
rs41542812	GG	CG	CC	P(GG VS. CC)	P(CG VS. CC)	CG+CC	P(CG+CCvs.CC)	GG	CG	CC	P(GG VS. CC)	P(CG VS. CC)	CG+CC	P(CG+CCvs.CC)
HDL	1.479±0.323	1.423±0.293	1.570±0.463	0.399	0.531	1.470±0.313	0.316	1.190±0.123	1.206±0.336	1.235±0.296	0.792	0.668	1.204±0.317	0.628
LDL	2.905±1.183	2.225±0.574	3.000±1.107	0.734	0.171	2.796±1.128	0.432	2.197±0.777	2.365±0.706	2.372±0.748	0.689	0.966	2.345±0.700	0.866
TG	1.332±0.751	1.373±0.967	1.275±0.994	0.806	0.848	1.339±0.766	0.770	1.103±0.203	1.471±0.677	1.693±1.579	0.521	0.518	1.427±0.648	0.409
TC	4.650±1.348	3.922±0.472	4.825±1.398	0.612	0.204	4.533±1.272	0.361	3.607±0.889	3.865±0.922	3.946±0.935	0.536	0.705	3.834±0.904	0.581
LDL/HDL	1.999±0.755	1.633±0.569	1.989±0.691	0.956	0.315	1.940±0.731	0.765	1.820±0.463	2.052±0.640	2.061±1.174	0.725	0.973	2.024±0.619	0.880
rs1049107	TT	CT	CC	P(TT VS. CC)	P(CT VS. CC)	TT+CT	P(TT+CT VS. CC)	TT	CT	CC	P(TT VS. CC)	P(CT VS. CC)	TT+CT	P(TT+CT VS. CC)
HDL	1.594±0.373	1.325±0.346	1.520±0.467	0.422	0.563	1.580±0.373	0.506	1.215±0.112	1.208±0.347	1.239±0.289	0.867	0.592	1.209±0.330	0.581
LDL	2.851±1.035	2.250±0.071	3.033±1.170	0.443	0.352	2.821±1.017	0.359	2.440±0.800	2.555±0.664	2.334±0.736	0.779	0.114	2.544±0.668	0.119
TG	1.478±1.027	0.815±0.191	1.187±0.875	0.144	0.554	1.444±1.011	0.187	1.113±±0.166	1.740±1.243	1.622±1.341	0.451	0.644	1.678±1.194	0.820
TC	4.741±1.246	3.738±0.455	4.791±1.458	0.864	0.316	4.689±1.235	0.723	3.878±0.905	4.110±0.858	3.898±0.923	0.966	0.227	4.088±0.854	0.262
LDL/HDL	1.833±0.651	1.750±0.410	2.078±0.725	0.100	0.530	1.829±0.638	0.087	1.980±0.495	2.218±0.672	2.024±1.224	0.943	0.368	2.194±0.655	0.406
rs1049100	TT	CT	CC	P(TT VS. CC)	P(CT VS. CC)	TT+CT	P(TT+CT VS. CC)	TT	CT	CC	P(TT VS. CC)	P(CT VS. CC)	TT+CT	P(TT+CT VS. CC)
HDL	1.553±0.420	1.280±0.410	1.548±0.443	0.955	0.405	1.580±0.373	0.932	1.215±0.112	1.208±0.347	1.239± 0.289	0.867	0.592	1.209±0.330	0.581
LDL	2.849±1.148	2.250±0.071	3.049±1.095	0.392	0.311	2.821±1.017	0.319	2.440±0.800	2.555±0.664	2.334± 0.736	0.779	0.114	2.544±0.668	0.119
TG	1.416±1.005	1.075±0.177	1.203±0.896	0.280	0.843	1.444±1.011	0.306	1.113±0.166	1.740±1.243	1.622± 1.341	0.451	0.644	1.678±1.194	0.820
TC	4.685±1.411	3.745±0.445	4.837±1.350	0.596	0.263	4.689±1.235	0.487	3.878±0.905	4.111±0.858	3.898± 0.923	0.966	0.227	4.088±0.854	0.262
LDL/HDL	1.903±0.742	1.840±0.537	2.039±0.673	0.355	0.682	1.829±0.638	0.334	1.980±0.495	2.218±0.672	2.024± 1.224	0.943	0.368	2.194±0.655	0.406
rs3891176	CC	AC	AA	P(CC VS. AA)	P(AC VS. AA)	CC+AC	P(CC+AC VS. AA)	CC	AC	AA	P(CC VS. AA)	P(AC VS. AA)	CC+AC	P(CC+AC VS. AA)
HDL	1.405±0.438	1.443±0.337	1.607±0.435	0.075	0.223	1.419±0.398	0.044	1.297±0.183	1.296±0.266	1.210±0.305	0.456	0.139	1.297±0.255	0.109
LDL	2.710±1.216	3.025±0.996	3.006±1.103	0.309	0.956	2.828±1.133	0.461	2.736±0.665	2.227±0.710	2.402±0.768	0.266	0.240	2.328±0.722	0.599
TG	1.128±0.638	1.091±0.389	1.380±1.072	0.322	0.361	1.114±0.551	0.191	1.523±0.864	1.360±0.870	1.794±1.712	0.680	0.154	1.387±0.871	0.151
TC	4.341±1.481	4.687±1.163	4.889±1.357	0.127	0.630	4.470±1.362	0.158	4.337±0.669	3.795±0.856	3.971±0.968	0.327	0.344	3.902±0.849	0.696
LDL/HDL	1.940±0.678	2.150±0.741	1.956±0.703	0.931	0.386	2.019±0.698	0.678	2.177±0.728	1.778±0.699	2.152±1.277	0.959	0.102	1.858±0.714	0.168

Supplementary Table 5. Analysis of plasma-lipid phenotypes and longevity-associated haplotypes.

		Longevity					Nonagenarians					Centenarians				
		normal	abnormal	P	OR	95%CI	normal	abnormal	P	OR	95%CI	normal	abnormal	P	OR	95%CI
HDL	CCC	86	19	-	-	-	66	14	-	-		20	5	-	-	-
	GTT	43	2	0.027	4.750	1.057-21.337	28	2	0.226	2.970	0.633-13.938	15	0	0.220	4.571	0.499-41.866
	CTT	28	0	0.045	6.667	0.857-51.883	22	0	0.112	5.149	0.644-41.173	6	0	1.000	2.000	0.204-19.618
LDL	CCC	63	42	-	-		50	30	-	-	-	13	12	-	-	-
	GTT	33	12	0.119	1.833	0.851-3.949	22	8	0.287	1.650	0.653-4.170	11	4	0.182	2.538	0.634-10.166
	CTT	20	8	0.380	1.667	0.672-4.133	18	4	0.089	2.700	0.835-8.736	2	4	0.654	0.462	0.071-2.994
TG	CCC	91	14	-	-		66	14	-	-	-					
	GTT	36	9	0.299	0.615	0.245-1.547	21	9	0.189	0.495	0.187-1.307					
	CTT	15	13	1.096×10^{-4}	0.178	0.070-0.451	11	11	0.016	0.259	0.090-0.743					
TC	CCC	67	38	-	-	-	50	30	-	-	-	17	8	-	-	-
	GTT	33	12	0.257	1.560	0.721-3.373	22	8	0.287	1.650	0.653-4.170	11	4	1.000	1.294	0.313-5.353
	CTT	18	10	0.963	1.021	0.428-2.436	16	6	0.374	1.600	0.565-4.535	2	4	0.174	0.235	0.035-1.564
LDL/HDL	CCC	67	38	-	-	-	34	46	-	-	-	6	19	-	-	-
	GTT	31	14	0.549	1.256	0.596-2.648	10	20	0.382	0.676	0.281-1.629	4	11	1.000	1.152	0.266-4.993
	CTT	24	4	0.027	3.403	1.098-10.544	4	18	0.037	0.301	0.093-0.969	0	6	0.648	0.408	0.042-3.932

Supplementary Table 6. Information of longevity and controls.

	LHAP			CLHLS			total		
	Nonagenarians	Centenarians	Control	Nonagenarians	Centenarians	Control	Nonagenarians	Centenarians	Control
Nos.	596	42	520	1928	253	2299	2534	295	2819
Mean Age(yr)	94.742±3.632	103.352±3.214	45.329±6.822	95.381±4.873	102.325±2.135	43.221±8.721	94.982±3.931	102.416±2.197	44.098±6.326
M:F	1：3.8	1：2.6	1：3.2	1:4.3	1:3.2	1:2.5	1:3.9	1:3.1	1:2.3

Supplementary Table 7. Primers of sequencing genotyping.

Varinats	Forward Primer	Reverse Primer	Product Length
rs41542812	TATCCCCTTACGCCACTCCA	ACTCTGGTCCAAGGAGGGAT	388bp
rs1049107	TATCCCCTTACGCCACTCCA	ACTCTGGTCCAAGGAGGGAT	388bp
rs1049100	TATCCCCTTACGCCACTCCA	ACTCTGGTCCAAGGAGGGAT	388bp
rs3891176	CCCCATGCTCACTTTGTCCT	CAGATCCATCAGGTCCGAGC	455bp
